# Does the sequence of rotations in Multiple Mini Interview stations influence the candidates’ performance?

**DOI:** 10.1080/10872981.2018.1485433

**Published:** 2018-06-18

**Authors:** Kyong-Jee Kim, Bum Sun Kwon

**Affiliations:** a Department of Medical Education, School of Medicine, Dongguk University, Goyang, South Korea; b Department of Rehabilitation Medicine, School of Medicine, Dongguk University, Goyang, South Korea

**Keywords:** Multiple Mini Interview, MMI, student selection

## Abstract

In Multiple Mini Interviews (MMIs), the candidates start out with different stations depending on the sequence of rotation they are assigned; thus, their perceived difficulty level and nervousness with their first station may differ. We examined whether such differences influenced the candidates’ overall performance in the MMI. A 32-item questionnaire was developed and administered to candidates for admission interviews at a medical school regarding their perceptions of the MMI. Candidates’ interview scores were also obtained and were compared across groups who differed in the sequence of rotation of MMI stations. Candidates felt nervous when they encountered the first station, which did not differ regardless of which station was their first encounter. Candidates were neutral regarding whether their first station was more difficult than the others and about whether its difficulty level influenced their overall performance in the test. Although candidates’ perceived station difficulty differed across stations, there were no differences in their performance in each station between those it was their first station and those it was not. Candidates’ total interview scores also did not differ across groups of different sequence of rotations. The MMI is a fair process as it does not give disadvantage to those who encounter a more difficult station than others for their first station.

## Introduction

In Multiple Mini Interviews (MMIs), which follow the format of Objective Structured Clinical Examinations (OSCEs) [1], the candidates encounter a series of interview stations. The candidates are given different scenarios in each MMI station and are asked to discuss their responses to the situations described in the scenario with the interviewer. MMIs are more valid and reliable than traditional interviews, particularly by using a multiple sampling approach and therefore have been adopted by many medical schools worldwide [1–14].

Although research has shown that the MMI process is fair [15], previous studies have indicated that the reliability of MMIs may vary widely depending on the way they are administered and structured [2,5]. Moreover, research indicates that examiner subjectivity and station difficulty would have affected the fairness of the test in MMIs [11,16]. In addition, there may be some personal factors that are related to the candidate’s performance in MMIs, such as cultural and social background, and his or her personality [10,17,18]. Therefore, more studies are warranted of factors that influence candidate performance in MMIs to improve the fairness of the test.

In particular, as there are multiple stations in MMIs, it is likely that the difficulty level of each station is heterogeneous. As the candidates rotate through in a coordinated sequence in the MMI, the first station that they encounter differs across candidates according to the sequence of rotation to which they are assigned randomly; hence, the difficulty level of their first station may differ. As research suggests that psychological factors influence a student’s performance in the test [19,20], the candidates’ perceived anxiety or nervousness of the MMI can potentially influence their performance in the test. Therefore, it would not be fair for some candidates to start with a more difficult station while others start with an easier one if that would affect their overall performance in the MMI. Nevertheless, there has been little research on the effects of the candidates perceived test difficulty of their first station on their performance in MMIs.

To remedy the potential problem of the impact of the random sequence of rotations among candidates on their overall performance in the MMI, one possible intervention would be to have all candidates rotate the stations in the same order. Yet, it takes more time to have candidates rotate the stations in the same order than when they rotate around the stations in different orders as in conventional OSCEs as some of the candidates can start the first station only and the others need to wait until the first group of candidates finish their first station and move on to the next for them to begin their first station. We at Dongguk University School of Medicine (DUMS) had adopted the format of having all candidates rotate the stations in the same order when we first implemented MMIs. Taking the time constraint when have candidates rotate stations in the same order into account, we needed to examine whether having them rotate stations in different sequences, not in the same order, would affect their overall performance in the test.

This study examined whether the candidates’ perceived anxiety, difficulty level of the MMI differ across candidates with different sequences of rotations in the MMI stations and whether such differences impact their performance in the test. The research questions were as follows:

1. Do the perceived difficulty and anxiety levels of candidates differ according to the first station that they encounter in the MMI?
2. Is there a difference in the candidates’ performance across the groups with different sequence of rotations in their interview stations?

## Methods

### Study setting and participants

This study was conducted on candidates for the admissions at DUMS, a private medical school in South Korea. The DUMS has a 4-year basic medical education program for graduate-entry students with an average class size of 50. The DUMS has implemented admission interviews for those who passed the initial screening stage based on their prior academic achievements, including undergraduate GPA and their performance in the Korean medical school entrance exam (the Medical Education Eligibility Test). In this specific year, 94 candidates were selected for the admission interviews according to the aforementioned criteria.

Since 2014, the admission interviews at DUMS have changed from the traditional format to MMIs. The MMI comprises six stations, in which candidates are presented with scenarios to assess their non-cognitive qualities identified by the school’s admission committee, which include self-regulation, communication skills, and ethical and logical reasoning. Each station is 10 min in duration and one interviewer is assigned to each station, who rates the candidates’ performance on a 5-point scale. Candidates’ performance in the MMI was assessed by cumulating their interview scores obtained in each station. Previous experience with the MMI at DUMS have shown that the MMI is a feasible tool for student selection [21].

### Study design and procedures

A semi-experimental study was implemented on interview candidates for entrance into the class of 2017, which was conducted in November, 2016. The candidates were divided into six groups with a different set of rotations of the MMI stations. A 32-item questionnaire was developed and administered to the candidates, which comprised of 7 items on the respondent demographics and 23 items on their perceptions of the MMI as well as two open-ended questions on their opinions of their experience with the MMI. The 23 items on the respondent overall perceptions of the MMI included eight items that were adapted from the post-MMI survey instrument developed by Eva et al. [1]. These items were translated into Korean by the authors and were piloted tested several times in previous years. The questionnaire also included five items on the candidates’ perceived anxiety and difficulty with the first station that they encountered and that of the overall test. These items were measured on a 5-point Likert scale, where 1 = ‘strongly disagree’ and 5 = ‘strongly agree’. Another six items measured the candidates’ perceived difficulty levels for each MMI station on a 5-point scale, where 1 = ‘very difficult’ and 5 = ‘very easy’.

The questionnaire was administered during the wrap-up session immediately after all the interviews had ended in the morning and afternoon sessions. Participation in the study was voluntary and consent was implied with the return of the survey as the responses were collected anonymously. An ethical review was conducted and informed consent was exempted by the institutional review board of Dongguk University, Gyeongju (IRB approval number: DGU IRB 2016001–01).

In addition to the survey data, the MMI scores were obtained and analysed to compare the candidate’s performance across the groups of different sets of rotations of the MMI stations. Descriptive statistics and analysis of variance (ANOVA) were performed to compare the candidates’ responses to the post-MMI survey and their overall performance across the groups of different sequences of rotations in the MMIs stations. A Mann–Whitney test was also performed to compare the candidate’s perceived station difficulty and their interview scores for each MMI station between those it was their first station and those it was not their first station. The data were analysed using SPSS version 23 for Windows (IBM Corp., Armonk, USA). The significance levels were 0.05 for statistical analysis.

## Results

### Participant demographics and backgrounds

A total of 91 candidates (53% male and 47% female) participated in the study, with a 100% response rate. Three of the candidates did not attend the interviews and were removed from the study. The mean age of the candidates was 27.1 years (SD = 3.42). The candidates’ backgrounds varied in terms of their undergraduate majors: 30% (*n* = 27) studied fields related to life science and biotechnology, 22% (*n* = 20) majored in engineering, 20% (*n* = 18) majored in sciences, 17% (*n* = 16) majored in health professions and 11% (*n* = 10) studied arts or humanities. Approximately two thirds (*n* = 58) of the candidates were from urban areas and one third (*n* = 33) were from rural areas.

### Participants’ perceived difficulty and anxiety levels across groups

Table 1 lists topics of the MMI stations and the results of the candidates’ responses on their perceived anxiety and difficulty level of those stations. The respondents agreed with the statement that ‘I was nervous when I encountered the first station’ (*M* = 4.03 ± .99), but there was no difference across the groups of candidates who differed in the station that they first encountered (*F* = 2.16, *p* = .07). The respondents also agreed with the statement that ‘Overall, I was nervous about the test,’ (*M* = 3.81 ± .88). On the other hand, there was no difference in candidates’ perceived overall anxiety regarding the test across the groups (*F* = 1.15, *p* = .34).

**Table 1.** Comparisons of candidates’ perceptions of the difficulty and nervousness of MMI stations across six groups who started with the same first station (*n* = 91).

Item	Mean * (SD)	F-value (p)
1. The first station was more difficult than the others.	3.12 (1.13)	1.54 (.19)
2. I was nervous when I encountered the first station.	4.03 (0.99)	2.16 (.07)
3. How nervous I was at the first station influenced my overall performance in the test.	3.33 (1.13)	.68 (.64)
4. The difficulty level of the first station influenced my overall performance in the test.	2.71 (1.07)	.31 (.90)
5. Overall, I was nervous about the test.	3.81 (0.88)	1.15 (.34)

* 1 = ‘strongly disagree’ 5 = ‘strongly agree’.

The respondents were neutral regarding the statement that ‘The first station was more difficult than the others’ (*M* = 3.12 ± 1.13), and there was no difference across the groups of candidates who differed in the station that they first encountered (*F* = 1.54, *p* = .19). The respondents were also neutral regarding the statement that ‘The difficulty level of the first station influenced my overall performance in the test’ (*M* = 2.71 ± 1.07), and there was no difference across the groups (*F* = .31, *p* = .90).

Table 2 shows the candidates’ perceived difficulty levels of MMI stations. The candidates’ perceived station difficulty levels of the test differed across stations, which ranged from *M* = 2.12 ± .80 to *M* = 3.06 ± .75 (1 = ‘very difficult,’ 5 = ‘very easy’). Candidates found stations #2 and 3 were the most difficult ones. The station #2 was about discussing outcomes of clinical trials and the station #3 dealt with a resident’s ethical dilemma in clinical practice. Moreover, candidates felt the extent to which each interview required specialized knowledge differed across stations. Candidates responded stations #2 and #4 required specialized knowledge the most, where *M* = 3.21 ± 1.10, and *M* = 3.19 ± 1.01, respectively (1 = ‘strongly disagree’ and 5 = ‘strongly agree’). In station #4 candidates were asked to discuss health outcomes data by applying basic science knowledge. Meanwhile, candidates found the station #1, which asked candidates’ past experiences, required the least specialized knowledge (*M* = 1.81 ± .91). Nevertheless, the candidates’ perceived difficulty level of each station did not differ across groups regarding whether it was their first station or not, as shown in Table 2.

**Table 2.** Comparisons of the candidates’ perceived station difficulty between those it was their first station and those it was not.

Station topics	Median *		Total Mean* (SD)	*Z* value (*p*)
	First station (*n* = 16)	Not first station (*n* = 75)		
1. Past experiences	3.00	3.00	3.06 (.75)	−.177 (.859)
2. Logical reasoning	2.00	2.00	2.12 (.83)	−.293 (.770)
3. Ethical problem	2.00	2.00	2.31 (.80)	−.400 (.689)
4. Logical reasoning	3.00	3.00	2.60 (.84)	−.145 (.884)
5. Critical thinking	3.00	3.00	2.59 (089)	−.247 (.805)
6. Communication skills	3.00	3.00	2.82 (.75)	−1.122 (.262)

* 1 = ‘very difficult’ 5 = ‘very easy’.

### Comparisons of the candidate performance across groups

Table 3 lists the differences in candidate performance in each MMI station across the groups. The candidates’ test scores did not differ among the groups regarding whether it was their first station or not. In particular, although candidates found the stations # 2 and 3 the most difficult as seen in Table 2, their interview scores in those two stations did not differ significantly between those it was their first station and those it was not their first station.

**Table 3.** Comparisons of the candidate performance in each MMI station between those it was their first station and those it was not.

	Median *			
Station topics	First station (*n* = 16)	Not first station (*n* = 75)	Total Mean* (SD)	*Z* value (*p*)
1. Past experiences	90	90	94.07 (5.57)	−.229 (.819)
2. Logical reasoning	90	90	92.74 (6.68)	−.440 (.159)
3. Ethical problem	80	90	87.14 (10.36)	−1.857 (.063)
4. Logical reasoning	90	90	89.01 (8.83)	−1.005 (.315)
5. Critical thinking	90	90	89.12 (9.02)	−.806 (.420)
6. Communication skills	90	90	90.00 (6.50)	−.848 (.397)

* interview score for each station (a possible 100 points per station).

Table 4 shows differences in candidates’ total interview scores across groups of different sequence of rotations in the MMI stations. There was no difference in the candidates’ total MMI scores across groups (*F* = 1.12, *p* = .36).

**Table 4.** Comparisons of the candidates’ total MMI scores across groups of different sequences of rotation in their MMI stations.

	Sum of squares	df	Mean square	*F*	*P*
Between group	2984.547	5	596.909	1.115	.359
Within group	45,518.750	85	535.515		
Total	48,503.297	90			

## Discussion

We found candidates felt nervous about the station that they encountered first in the MMI. It is likely that candidates were nervous about the MMI because it was a high-stake test and they were highly motivated to gain entry into medical school. Still, candidates are likely to be less nervous as they become accustomed to the format of MMI as they move onto the next stations as they were neutral regarding whether such nervousness influenced their overall performance in the test. Our study confirms the speculation that the candidates’ perceived difficulty of the test differ across stations in the MMI. Still, there were no difference in candidates’ overall perceptions of the difficulty and anxiety of the test across the six groups who differed in the sequence of rotations of the MMI stations.

There was a difference in the median of the interview scores in one station between those it was their first station and those it was not, although the difference was not statistically significant. In this station, candidates were asked to respond to an ethical dilemma in a clinical situation. Candidates responded that this specific station was the second most difficult one, and was also one of the stations that required specialized knowledge the most. Therefore, it can be speculated that this station was somewhat more difficult and unfamiliar for them than the others as it dealt with a clinical situation. This may have affected performance of those who it was the first stations for them particularly. Nevertheless, the candidates perceived that the difficulty level of their first station did not likely influence their performance for the remaining stations, and there was no difference in their overall performance in terms of the total MMI scores across the groups of different sequence in the rotation of the stations.

Our findings confirm the fairness of the random sequence of stations in the MMI in that it does not disadvantage those who encounter a more difficult station first than others. Although candidates felt somewhat nervous about the test when they encounter a somewhat difficult first station, it did not affect their overall performance in the test. This study supports the claims made by previous studies regarding the strengths of the MMI in that it offers candidates with opportunities to recover from the station that they do not do well at the other stations and the candidate disadvantaged at one station may not result in similar disadvantages at the other stations [1,5]. It was our previous experience from traditional panel interviews implemented prior to the adoption of MMIs that candidates had rarely recovered from a station that was unsatisfactory for them because there were only a few interview questions.

This study has some practical implications for implementing MMIs. First, the study confirmed the fairness of the format of the random sequence of stations in MMIs as it does not influence their overall performance in the test. Second, as a majority of candidates agreed that they were nervous when they encountered their first station, the assessor needs to take this into consideration when he or she interacts with them for whom it is their first station and make them feel comfortable about the interview.

This study had some limitations. First, the sample size of the experimental group for each station, that is those for whom it was their first station, was small with 16 candidates per station. Therefore, further study with a larger sample will be needed to enhance the generalizability of this study. Second, this study assessed respondent feelings in an ordinal scale using the Liker-type assessment. Some researchers argue respondent attitudes need to be measured as interval data because one cannot assume that the distance between responses is equivalent [22,23]. Future study is warranted for more in-depth understanding of candidate perceptions by using a qualitative research method to enhance the validity of our findings. Third, although participation in this study was voluntary, all candidates completed the questionnaires. Such a high response rate can potentially be a study limitation. The candidates were reminded that their responses to the questionnaires would not affect the results of their interviews. Yet, they participated in the survey just after the interviews; therefore, they might have been more cooperative to the study and tended to respond in more favourable ways, which may cause response bias [24]. Still, it is not clear from this study how this affects the results of the study. Future study is warranted to analyse candidate perceptions by using more various sources of data. Fourth, there may other factors that influence the fairness of the test in the MMI in addition to the sequence of stations such as interviewer subjectivity, and the candidate’s cultural and social background, and his or her personality, which were previously mentioned. Further study on other aspects of the MMI that may affect the fairness is warranted to enhance the fairness of MMIs.

## Conclusions

Our study offers empirical evidence of the fairness of the random sequence of stations in the MMI in that it does not disadvantage those who encounter a more difficult station first than others.
